# Pulmonary embolism and aseptic meningitis complicating IVIG for common variable immunodeficiency syndrome

**DOI:** 10.1016/j.jacig.2026.100666

**Published:** 2026-02-13

**Authors:** Daniel Butz, Arun Adlakha

**Affiliations:** a Brooke Army Medical Center, Fort Sam Houston, Tex; b Edward Via College of Osteopathic Medicine (VCOM)-Carolinas, Spartanburg, SC; c Carolina Lung Clinic, Spartanburg, SC

**Keywords:** Common variable immunodeficiency management, intravenous immunoglobulins, IVIG, recurrent aseptic meningitis, acute pulmonary embolism, common variable immunodeficiency

## Abstract

This case highlights the importance of vigilance for both thrombotic and inflammatory adverse events in patients with common variable immunodeficiency who are receiving intravenous immunoglobulin. It demonstrates that a multifaceted approach, including altering the product, infusion rate, and premedication, can effectively manage these complex complications.

Common variable immunodeficiency (CVID) is a primary immunodeficiency disorder characterized by impaired B-cell differentiation and defective immunoglobulin production.^1^ It is the most common primary immunodeficiency in adults, with an estimated prevalence of 1 in 10,000 to 1 in 100,000.^2^ CVID clinical manifestations vary, but recurrent respiratory tract infections are common.^3^ Diagnosis relies on clinical presentation, laboratory findings, and exclusion of other immunodeficiencies.^1^ Intravenous immunoglobulin (IVIG) therapy is a cornerstone of CVID management, providing exogenous antibodies to prevent infections.^3^ However, IVIG treatment carries a risk of adverse events, including thrombotic events with an estimated incidence between 1% and 16.9%.^4^^,^^5^ Additionally, IVIG therapy carries a risk for aseptic meningitis, with an estimated incidence between 0.6% and 1.0%.^6^^,^^7^

## Case presentation

A 59-year-old White female with a 3-year history of recurrent severe infections, including gram-negative bacilli, methicillin-resistant *Staphylococcus aureus*, and fungal sepsis, presented for evaluation. After other causes had been ruled out, a diagnosis of CVID was made on the basis of low total IgG level, low levels of IgG subclasses 2 and 3, low IgM level, and a nonresponsive qualitative antibody response to pneumococcal vaccination.

In fall 2017, the patient began IVIG therapy with intravenous human immune globulin 10% (Privigen, CSL Behring, King of Prussia, Pa), 400 mg/kg every 3 weeks administered over 3.5 hours. Premedication included oral diphenhydramine and intravenous methylprednisolone, along with preinfusion and postinfusion intravenous fluids. Initially, this therapy was well tolerated, and it reduced the patient’s infection frequency and severity.

Approximately 4 years into stable IVIG therapy, the patient was hospitalized for acute right-sided pleuritic chest pain and worsening dyspnea, occurring about 10 days after her most recent infusion. A computed tomography angiogram of the chest revealed an acute pulmonary embolism (PE) in the right interlobar artery and segmental branches. The results of a thorough workup for other pulmonary embolism PE risk factors—including inherited or acquired hypercoagulable states, obesity, varicose veins, and prolonged immobility—were negative, implicating IVIG as the probable cause. Her trough IgG level at the time was not measured, but her levels were regularly maintained within the therapeutic range. She was treated with anticoagulation.

Within 48 hours of an infusion performed 2 months later, she presented with headache, neck stiffness, nausea, photophobia, and fever. Lumbar puncture showed neutrophilic pleocytosis and elevated protein level. The results of comprehensive infectious workups, including bacterial cultures and viral PCR panels, were negative. She was diagnosed with IVIG-induced aseptic meningitis. One month later, she experienced a recurrence of the identical meningeal symptoms within 48 hours of infusion.

Following a neurology evaluation, her regimen was modified, as she preferred to continue intravenous therapy. She was switched to a different product (Gamunex-C 10%, Grifols, Clayton, NC), administered every 3 weeks at a lower dose (350 mg/kg) over a slower infusion time (5 hours). Premedication was intensified with a higher dose of intravenous methylprednisolone and the addition of intravenous promethazine. Prophylactic enoxaparin (administered subcutaneously twice daily) was initiated on the day of infusion and continued for 7 days after infusion. Since these changes, she has had no further episodes of PE or aseptic meningitis.

## Discussion

This case is unique, as it presents a patient with CVID who was experiencing both PE and recurrent aseptic meningitis in close temporal proximity to IVIG therapy.

First, there is no definitive evidence that the clinical features of IVIG-induced complications in patients with CVID are distinct from those in patients with other conditions. However, the underlying immune dysregulation in CVID could theoretically alter a patient's susceptibility.

Second, the delayed onset of complications after 4 years of stable therapy is notable. This may suggest a cumulative effect or a "second hit" phenomenon, in which an unidentified transient factor (eg, minor illness, dehydration) lowered her tolerance to the infusion. The 10-day gap between the infusion and the PE is also longer than typically reported but is mechanistically plausible. IVIG-induced hyperviscosity, activation of procoagulant factors (including factor XIa, and endothelial inflammation) can initiate a prothrombotic state that may not manifest as a clinical event for several days.^8^

Finally, because multiple interventions were made simultaneously, it is difficult to isolate the most influential factor in preventing recurrence. However, slowing the infusion rate and changing the IVIG product were likely the most critical modifications. Slower infusion rates are a well-established method to reduce adverse reactions, and different IVIG formulations contain varying stabilizers and levels of IgG aggregates, which can influence inflammatory and procoagulant responses.^9^

The successful management of these complications underscores the importance of modifying the infusion regimen when adverse events occur. The addition of prophylactic subcutaneous enoxaparin for venous thromboembolism prevention during the week of infusion is a noteworthy strategy for high-risk patients who prefer infusion over continuous daily oral anticoagulation.

Ethical considerations: Informed consent was obtained from the patient to publish this case report. Given the retrospective nature of this case report, there were no ethical considerations

Declaration of artificial intelligence (AI) and AI-assisted technologies in the writing process: During the preparation of this work, the authors used the Google AI platform Gemini to ensure accurate editing and revision of article. After using this tool/service, the authors reviewed and edited the content as needed and take full responsibility for the content of the publication.

## Disclosure statement

Disclosure of potential conflict of interest: The authors declare that they have no relevant conflicts of interest. The views expressed in this article are those of the authors and do not necessarily reflect the official policy or position of the Defense Health Agency, the Department of Defense, or any agencies of the US Government.
